# Watering Horses

**Published:** 1881-05

**Authors:** 


### WATERING HORSES.

If after feeding hay and then oats we allow a horse to take a large drink of water, a considerable part of the oats will be carried by the water into the intestines, and he get little of the advantage of feeding the oats after the hay. If such a drink is taken soon after eating hay alone, the effect will not be so injurious, because hay does not need so long a time for digestion as grain. If only one or two quarts of water are allowed, it will pass the food in the stomach without changing its position to any great extent. When the stomach has got rid of a considerable portion of its contents, it seems a difficult matter for it to force out the remainder, and fermentation and colic sometimes result. A drink of water at such a time, by carrying on the substance which has remained long enough, relieves the condition. This probably explains why some horse-car companies have found it advisable to have their horses watered at midnight.

